# Supplementary material for: Effect of New Thiophene-Derived Aminophosphonic Derivatives on Growth of Terrestrial Plants. Part 2. Their Ecotoxicological Impact and Phytotoxicity Test Toward Herbicidal Application in Agriculture
Source: Molecules. 2018 Dec 1;23(12):3173. doi: 10.3390/molecules23123173 (PMC6321426; doi:10.3390/molecules23123173)
Supplement: Supplementary file 1 [file molecules-23-03173-s001.pdf]

Effect of new thiophene-derived aminophosphonic derivatives on growth of terrestrial plants. Part 2. Their phytotoxicological tests on popular weeds and their ecotoxicological impact.

Diana Rogacz [1], Jarosław Lewkowski [2,*], Zbigniew Malinowski [2], Agnieszka Matusiak [2], Marta Morawska [2] and Piotr Rychter [1,*]

[1] Faculty of Mathematics and Natural Science, Jan Długosz University in Częstochowa, 42-200 Częstochowa, 13/15 Armii Krajowej Av., Poland; diana.rogacz@gmail.com (D.R.);

[2] Department of Organic Chemistry, Faculty of Chemistry, University of Łódź, Tamka 12, 91-403 Łódź, Poland; zbigniew.malinowski@chemia.uni.lodz.pl (Z.M.); agusk@poczta.fm (A.M.); mz.morawska@gmail.com (M.M.)

* Correspondence: (chemistry) jlewkow@uni.lodz.pl; Tel.: +48-42-635-5751

* Correspondence: (biology) p.rychter@ajd.czest.pl; Tel.: +48-34-361-5154

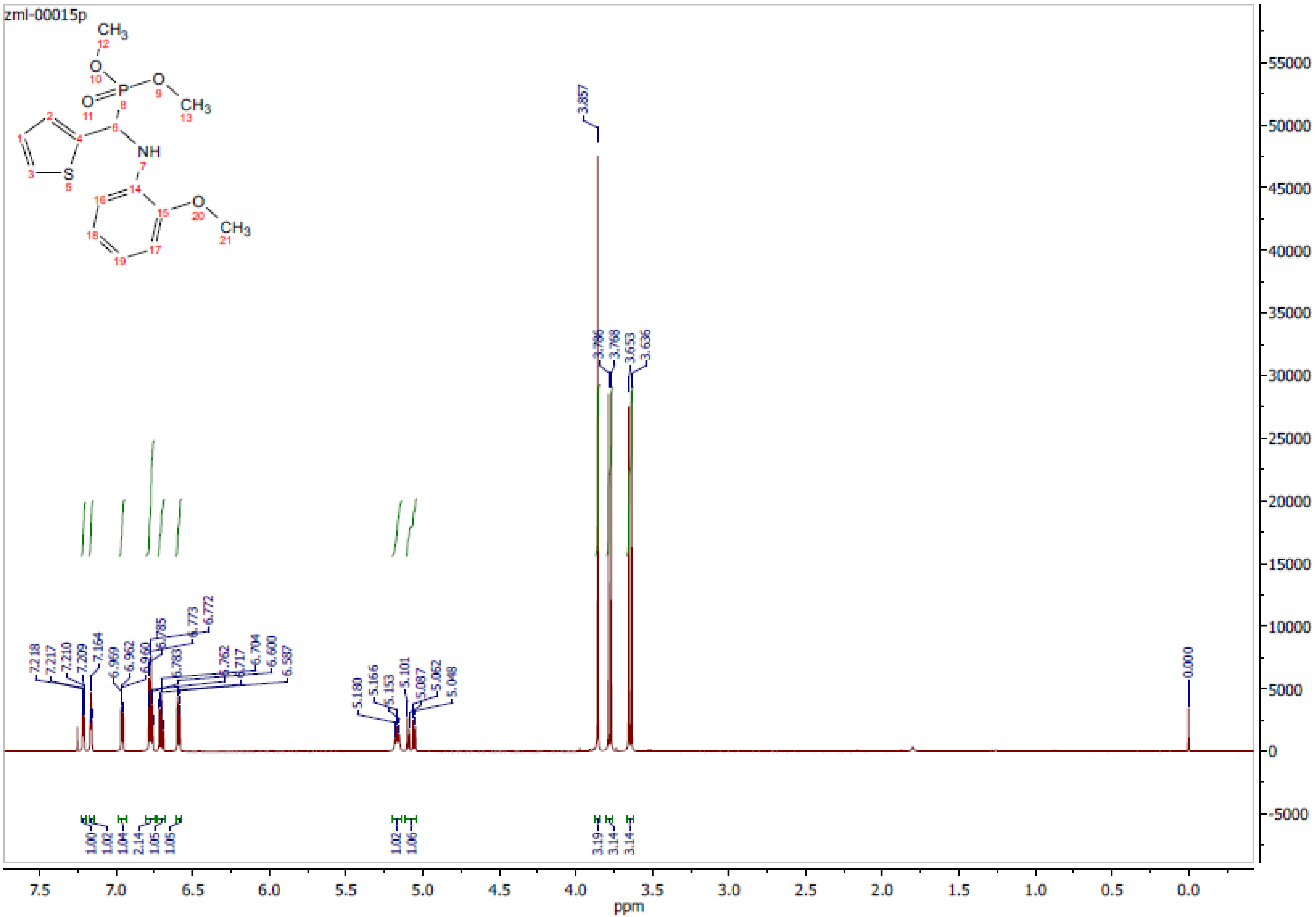

Fig. S1. ^{1}H NMR spectrum of dimethyl N-(2-methoxyphenyl)amino(2-thienyl)methylphosphonate (2d)

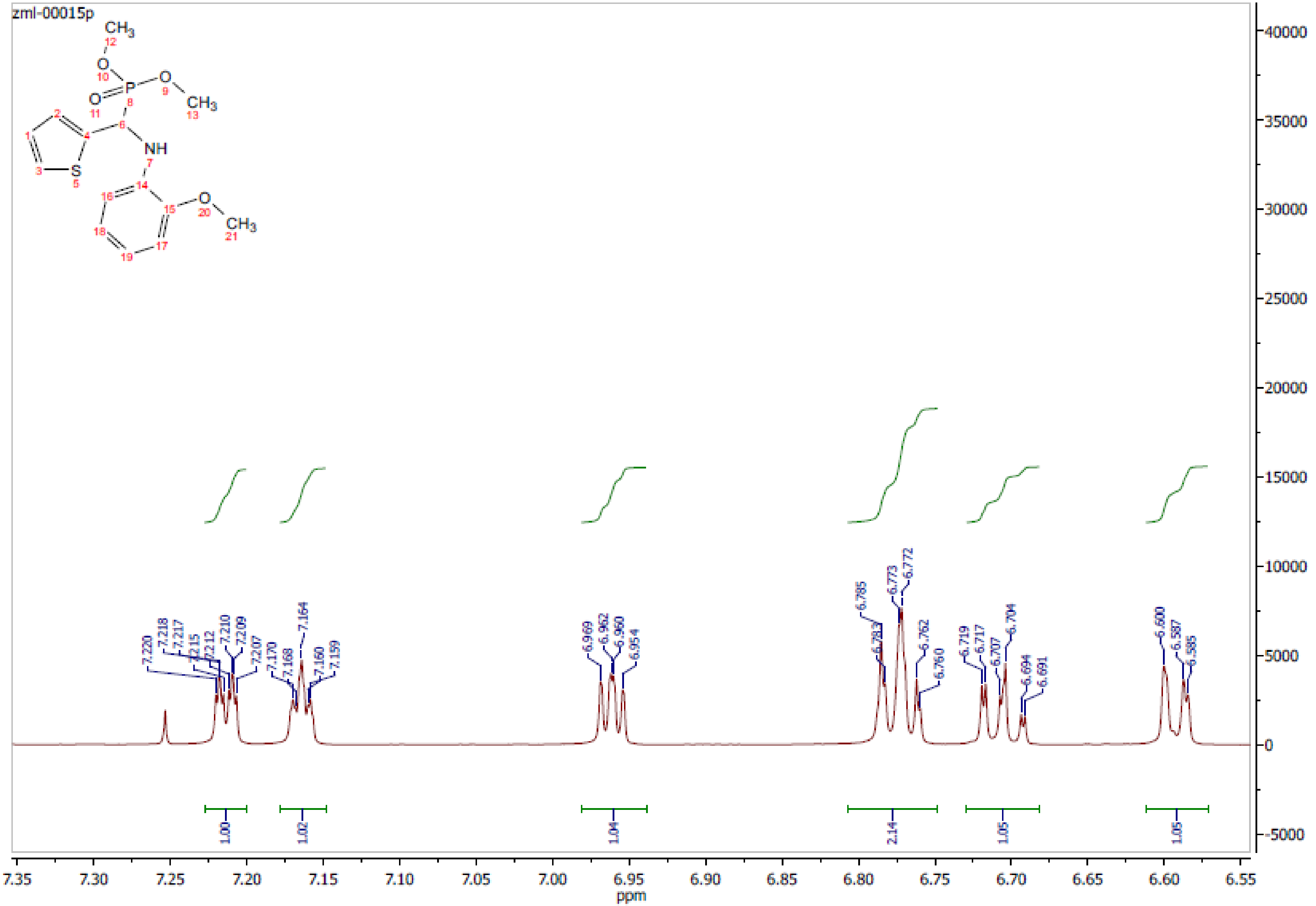

Fig. S2. ^{1}H NMR spectrum of dimethyl N-(2-methoxyphenyl)amino(2-thienyl)methylphosphonate (2d) range 7.5-6.5 ppm

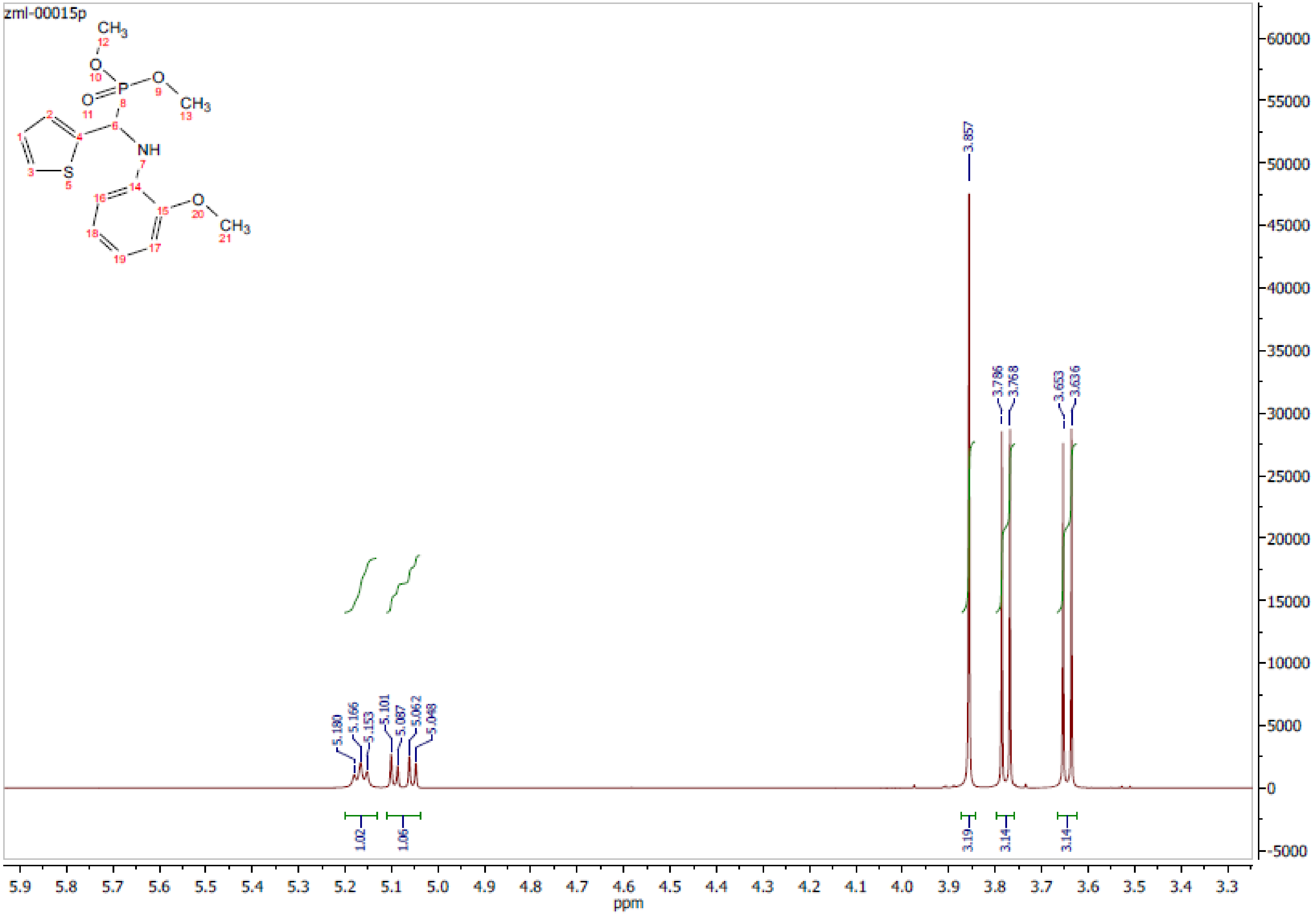

Fig. S3. **^{1}H NMR spectrum of dimethyl N-(2-methoxyphenyl)amino(2-thienyl)methylphosphonate (2d) range 6.0-3.3 ppm**

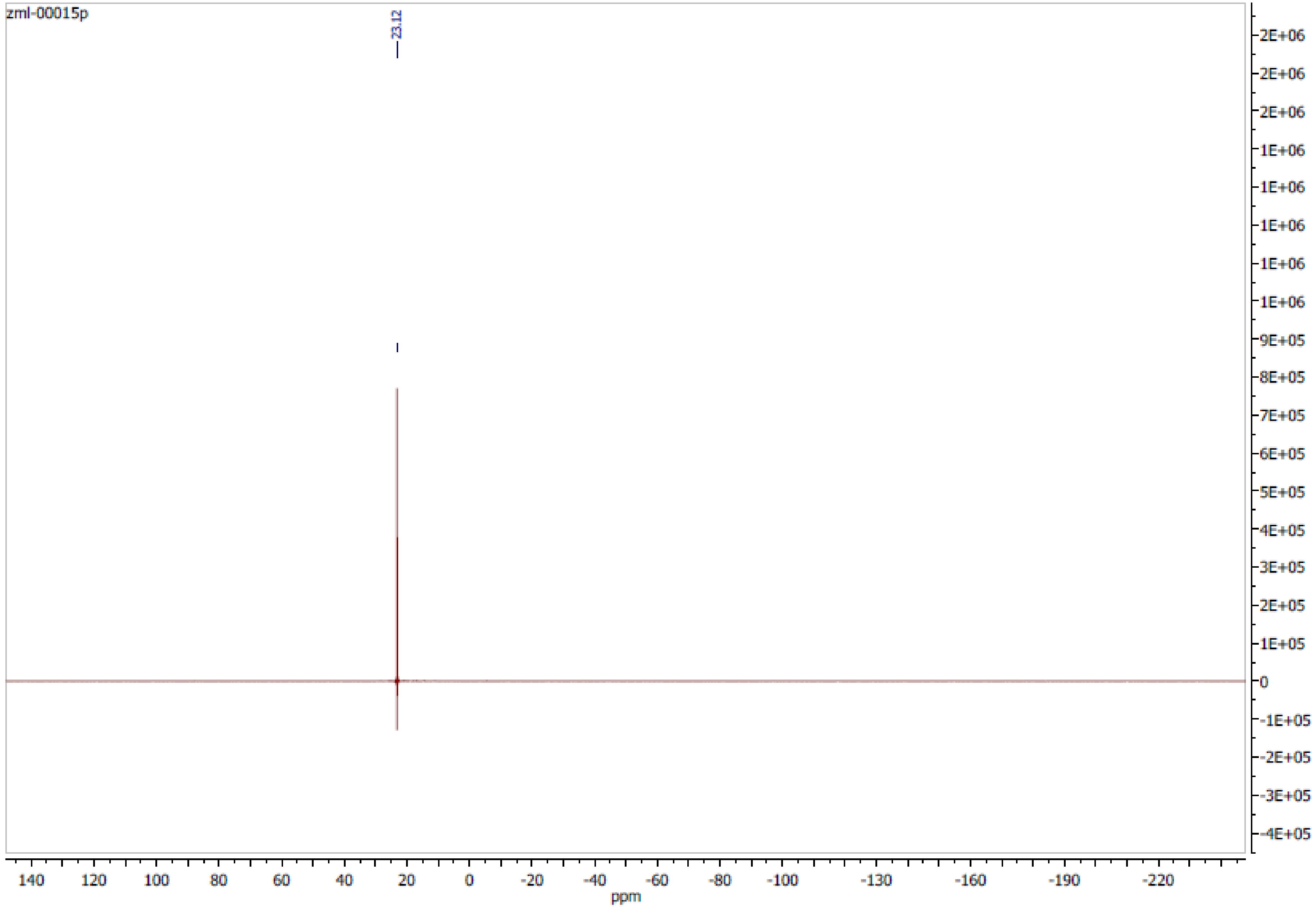

Fig. S4. ^{31}P NMR spectrum of dimethyl N-(2-methoxyphenyl)amino(2-thienyl)methylphosphonate (2d)

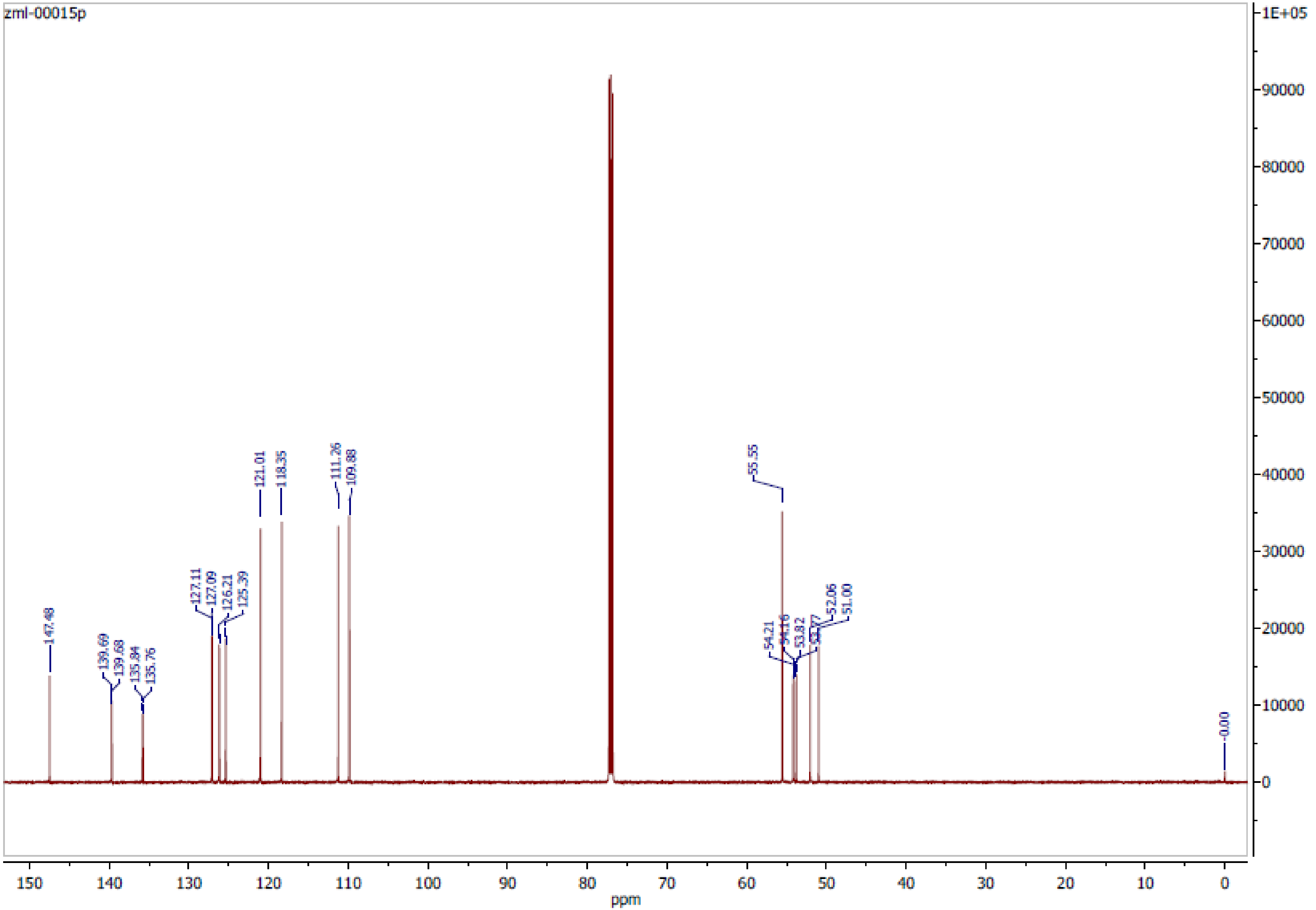

Fig. S5. ^{13}C NMR spectrum of dimethyl N-(2-methoxyphenyl)amino(2-thienyl)methylphosphonate (2d)

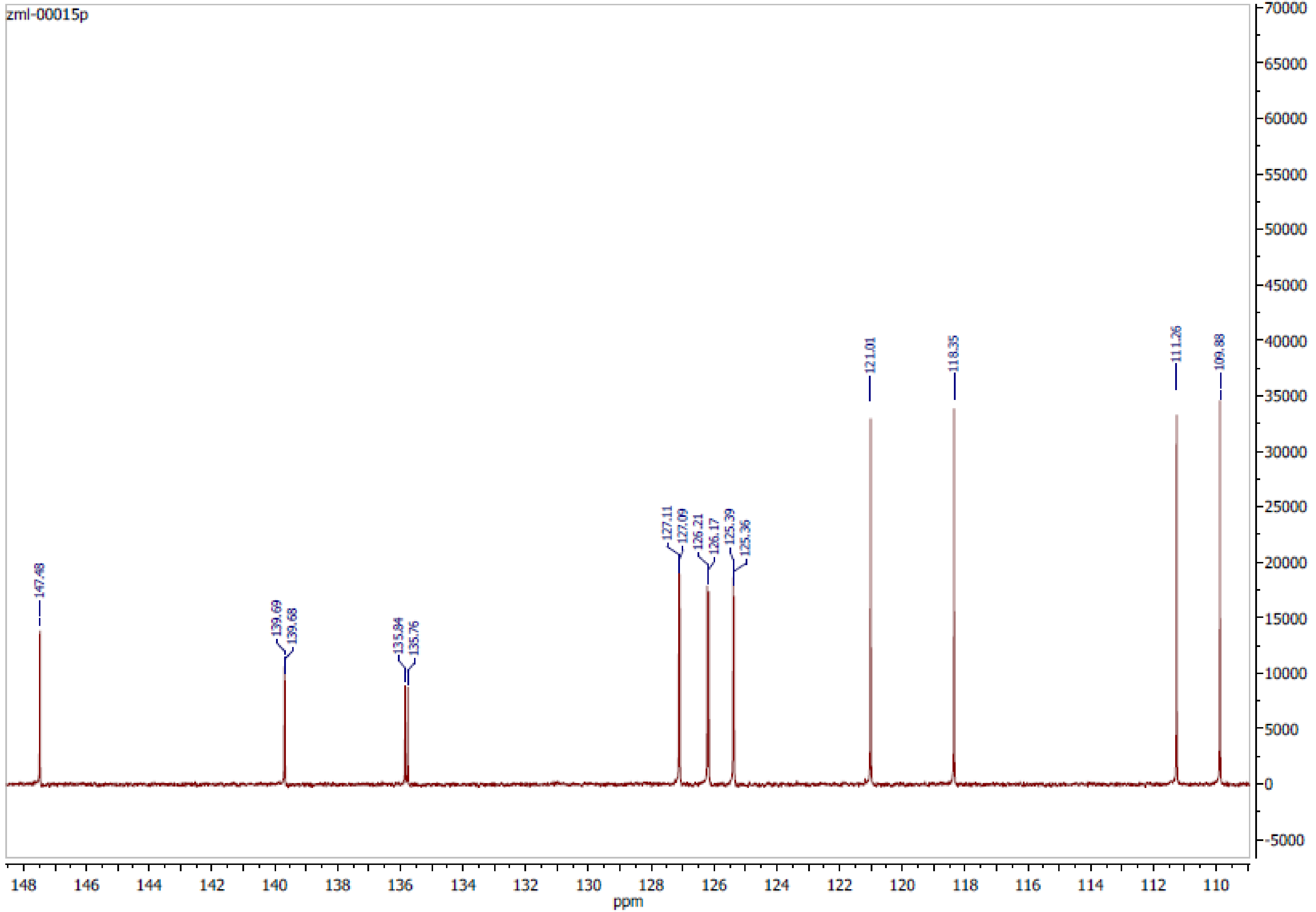

Fig. S6. ^{13}**C NMR spectrum of dimethyl N-(2-methoxyphenyl)amino(2-thienyl)methylphosphonate (2d) – range 148-109 ppm**

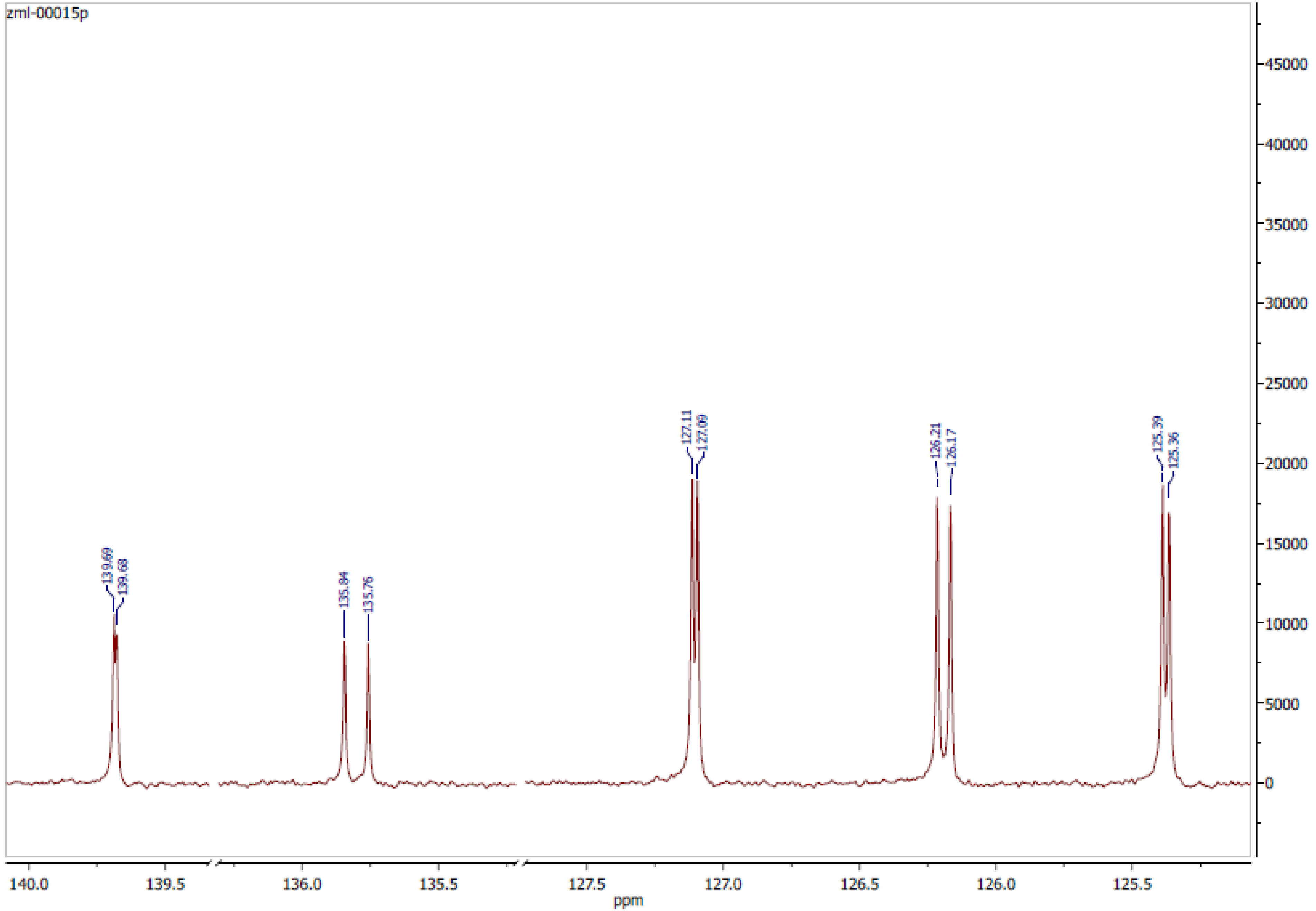

Fig. S7. ^{13}C NMR spectrum of dimethyl N-(2-methoxyphenyl)amino(2-thienyl)methylphosphonate (2d) – range 140-125 ppm

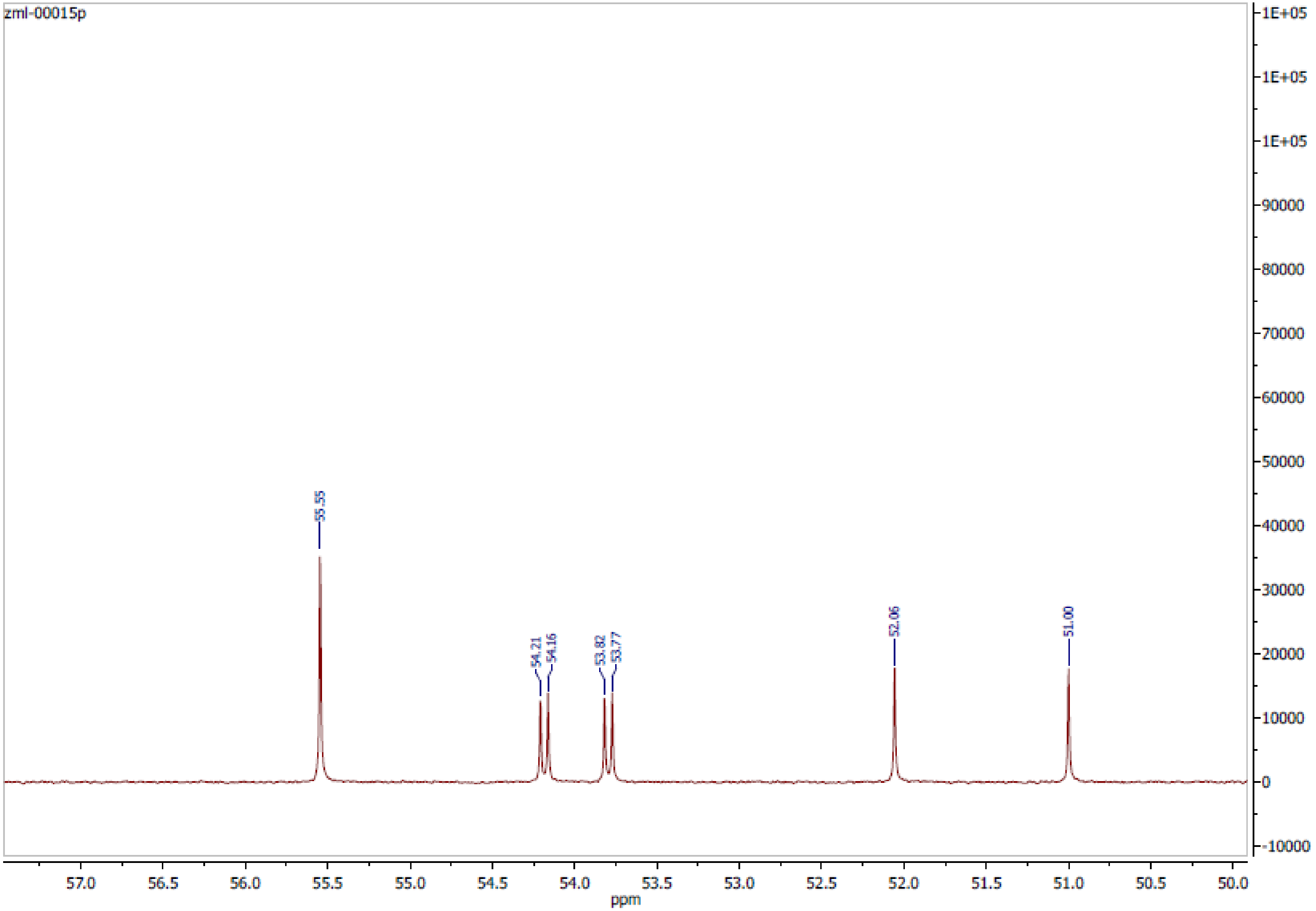

Fig. S8. ^{13}C NMR spectrum of dimethyl N-(2-methoxyphenyl)amino(2-thienyl)methylphosphonate (2d) – range 58-50 ppm

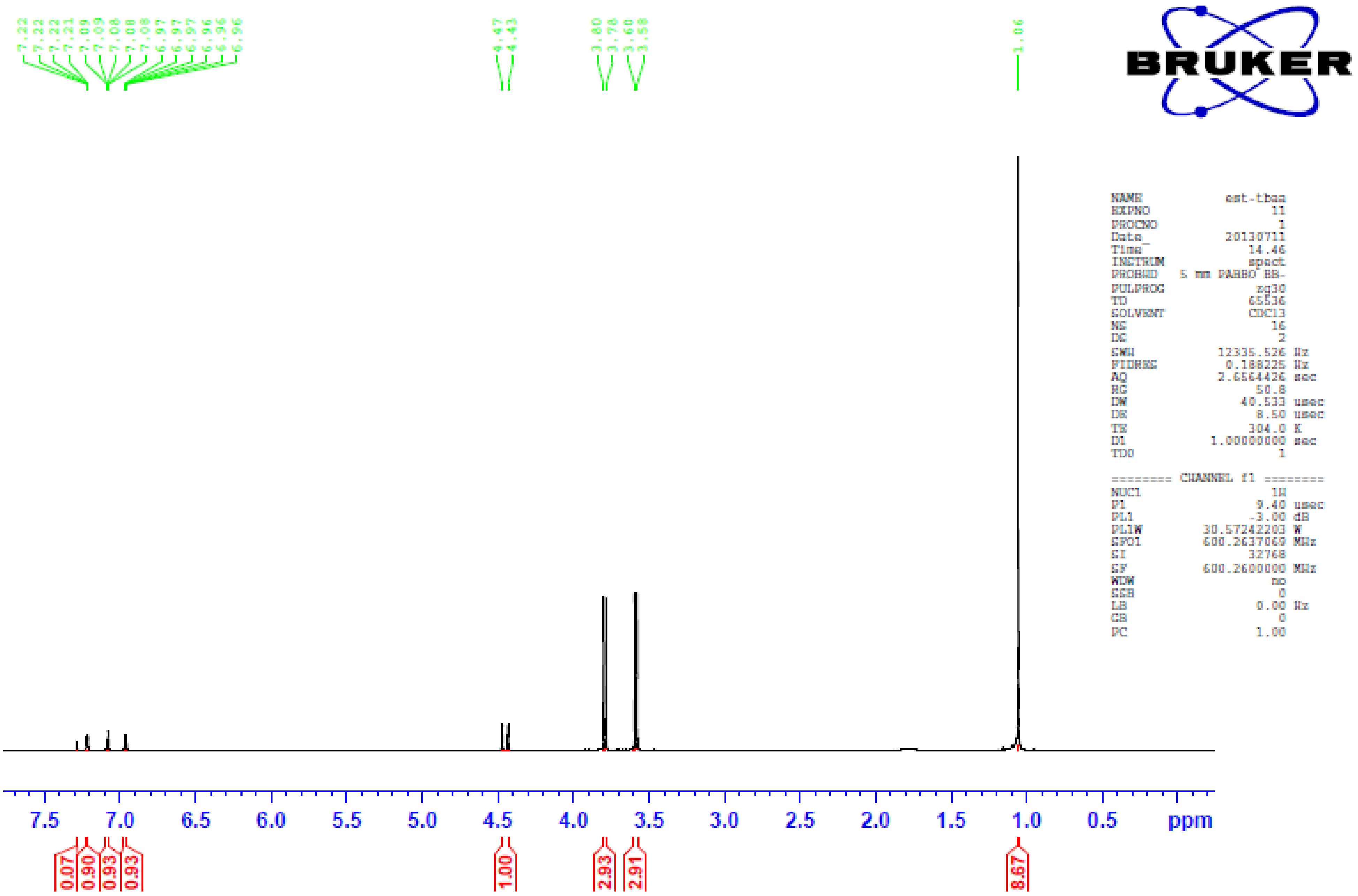

Fig. S9. ^{1}H NMR spectrum of dimethyl N-(t-butyl)amino(2-thienyl)methylphosphonate (2h)

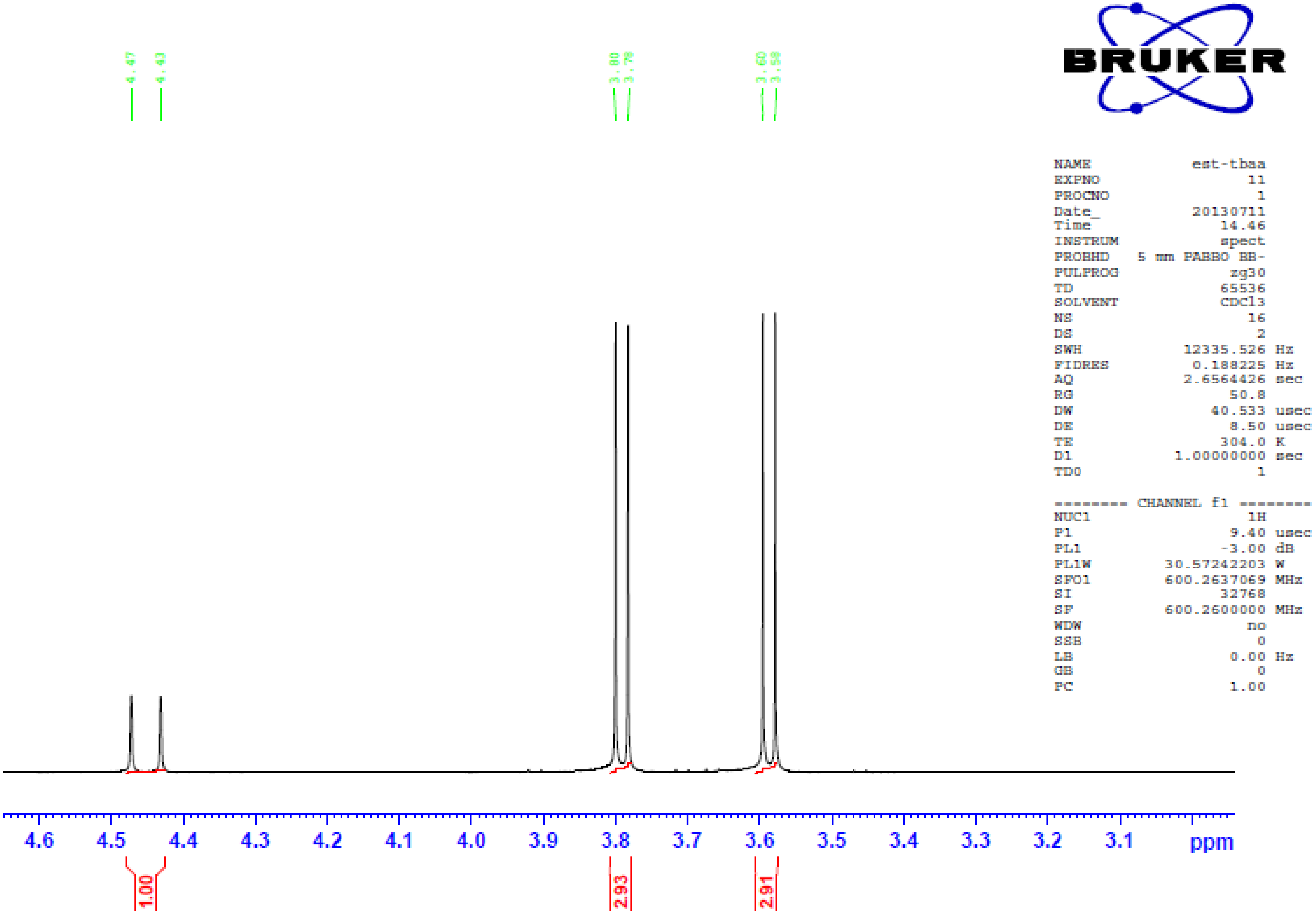

Fig. S10. ^{1}H **NMR spectrum of dimethyl N-(t-butyl)amino(2-thienyl)methylphosphonate (2h) – range 4.6-3.0 ppm**

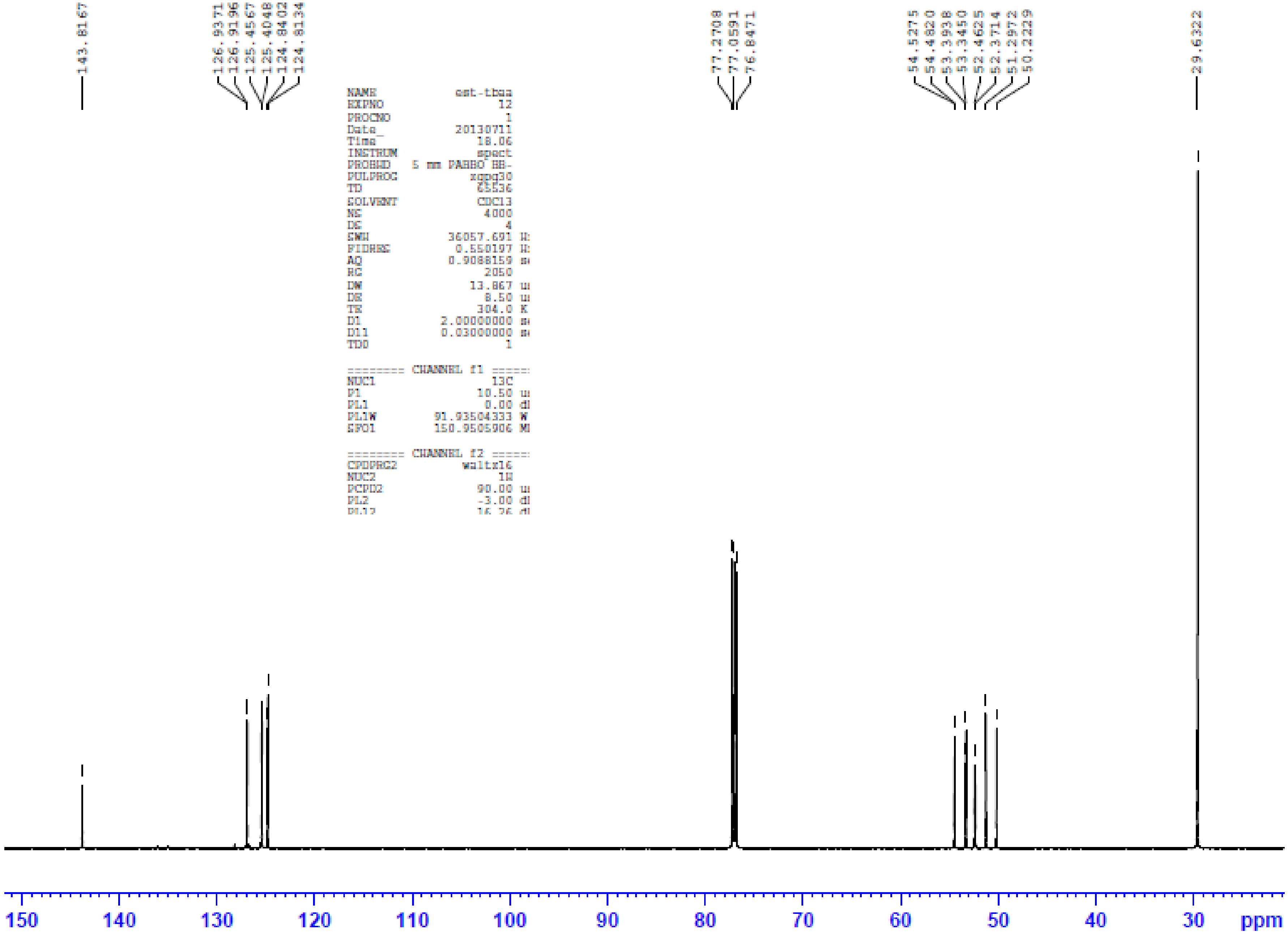

Fig. S11. ^{13}C **NMR spectrum of dimethyl N-(t-butyl)amino(2-thienyl)methylphosphonate (2h)**

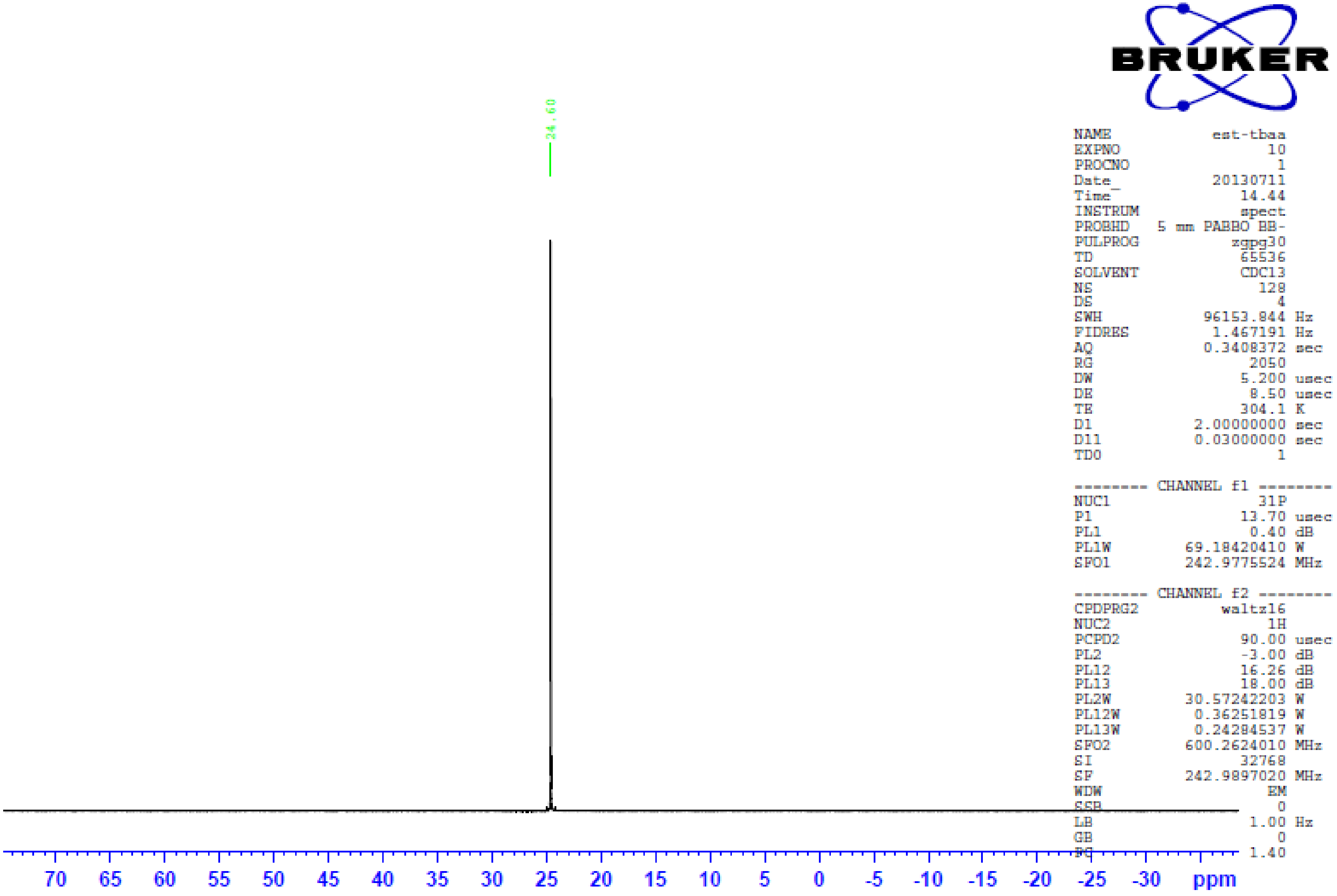

Fig. S12. ^{31}P NMR spectrum of dimethyl N-(t-butyl)amino(2-thienyl)methylphosphonate (2h)
